# Effects of Diminished Cerebrospinal Fluid Flow in the Spinal Canal on Amyloid Pathophysiology in Vervet Monkeys

**DOI:** 10.21203/rs.3.rs-6551242/v1

**Published:** 2025-05-13

**Authors:** Jeongchul Kim, Megan E. Lipford, Richard A. Barcus, Brett M. Frye, Hongyu Yuan, Qing Lyu, Jeremy P. Hudson, Samuel N. Lockhart, Courtney L. Sutphen, Thomas C. Register, Michelle M. Mielke, Suzanne Craft, Carol A. Shively, Christopher T. Whitlow

**Affiliations:** Department of Radiology, Wake Forest School of Medicine; Department of Radiology, Wake Forest School of Medicine; Department of Radiology, Wake Forest School of Medicine; Department of Biology, Emory and Henry University; Department of Radiology, Wake Forest School of Medicine; Department of Radiology, Wake Forest School of Medicine; Department of Radiology, Wake Forest School of Medicine; Division of Geriatric Medicine, Department of Internal Medicine, Wake Forest School of Medicine; Wake Forest Alzheimer’s Disease Research Center, Wake Forest School of Medicine; Wake Forest Alzheimer’s Disease Research Center, Wake Forest School of Medicine; Department of Epidemiology and Prevention, Wake Forest School of Medicine; Division of Geriatric Medicine, Department of Internal Medicine, Wake Forest School of Medicine; Department of Pathology, Section on Comparative Medicine, Wake Forest School of Medicine; Department of Radiology, Wake Forest School of Medicine

**Keywords:** Cerebrospinal Fluid, Beta-Amyloid, Magnetic Resonance Imaging, Clearance, Alzheimer’s disease, Vervet, African Green Monkey

## Abstract

**Background:**

Translational models using nonhuman primates provide valuable insights into the pathological changes associated with human brain aging. This study investigates the vervet monkey (*Chlorocebus aethiops sabaeus*) as a model for age-related Alzheimer’s disease (AD)-like amyloid clearance impairment. The cerebrospinal fluid (CSF) plays a crucial role as a transport medium for waste clearance from the brain’s interstitial space to lymphatic vessels.

**Methods:**

To test the hypothesis that diminished CSF perfusion in the central nervous system contributes to AD pathophysiology, we quantified CSF flow dynamics (i.e. stroke volume, amplitude and peak flow) in the spinal canal via phase-contrast MRI and examined its association with age and AD fluid biomarkers in 16 female vervet monkeys aged from 10 to 27 years.

**Results:**

Strong negative correlations were observed between CSF flow metrics across the cardiac cycle and age (ρ = −0.68 to −0.83), and positive correlations were found between CSF flow metrics and CSF Aβ42/40 ratio (ρ = 0.67 to 0.84), while plasma biomarkers showed no correlation with CSF flow metrics. Age-adjusted analyses demonstrated moderate correlations between CSF flow and CSF Aβ42/40 ratio (ρ = 0.45 to 0.75).

**Conclusion:**

These findings suggest that diminished CSF flow dynamics in the central nervous system could serve as a valuable imaging marker for impaired amyloid clearance, reflecting early-stage AD-like pathology.

## Background

An estimated 6.9 million Americans aged 65 and older are living with Alzheimer’s Disease (AD) in 2024. This number could grow to 13.8 million by 2060 [1], barring the development of medical breakthroughs to prevent or cure AD. A defining characteristic of AD is the accumulation of amyloid-beta (Aβ) and tau, which is thought to result from an imbalance between their production and clearance in the brain [2, 3]. Recent evidence suggests that this imbalance is primarily driven by impaired clearance rather than increased Aβ and tau production [4–6]. Various mechanisms contribute to this clearance, including enzymatic degradation, cellular uptake, and transport across the blood-brain barrier (BBB) [7] and blood-cerebrospinal fluid barrier. Additionally, interstitial fluid (ISF) bulk flow [4] and cerebrospinal fluid (CSF) absorption into the circulatory and lymphatic systems [8] play significant roles. Another key process, intramural peri-arterial drainage (IPAD) [9], works in conjunction with BBB transport, pericyte-mediated degradation, and other clearance pathways to regulate Aβ levels. While these studies have been informative, much of the evidence also comes from invasive fluorescent microscopy studies in small animals, which are not directly applicable to in vivo clearance measurements in the human brain. Therefore, there is the urgent need for non-invasive biomarkers to characterize clearance function in the brain, bridging the gap between preclinical findings and their translation into clinical practice.

Recent research emphasizes the crucial role of CSF circulation in the subarachnoid space as a key component of the brain’s clearance mechanisms, where it serves as a transport medium [4, 9–12]. The brain’s waste clearance system relies on the glymphatic system and IPAD, where CSF flows through perivascular spaces to flush out interstitial waste. The collected waste in the subarachnoid space is then transported into meningeal lymphatic vessels, emphasizing the critical role of efficient CSF circulation in delivering waste to the broader lymphatic network and highlighting the interplay between CSF flow and lymphatic pathways. As brain degeneration progresses, CSF production from the choroid plexus declines, while the lateral ventricles and subarachnoid space expand to compensate for volumetric loss due to atrophy [13]. This expansion, however, leads to a reduced CSF turnover rate, and circulation within the subarachnoid space may become disrupted [14, 15]. CSF flow dynamics within the central nervous system are likely the fundamental driving force behind CSF clearance. Thus, understanding the relationship between CSF flow dynamics and Aβ pathophysiology in the aging brain is essential for developing early diagnostic tools and therapeutic interventions for AD.

Animal models for AD are essential tools for understanding the disease’s mechanisms and for developing methods of prevention, diagnosis, and treatment [16–20]. Nonhuman primates (NHPs) serve as valuable models of neurocognitive aging and are particularly useful for studying the impacts of hypertension, reduced gait speed, sarcopenia, and glucose intolerance [21–24]. Vervets (*Chlorocebus aethiops sabaeus*) are advantageous as AD models due to their close phylogenetic proximity to humans, including similarities in brain structure and function, as well as comparable endocrine, social, and cognitive characteristics [25]. Their larger body size also facilitates imaging studies and CSF collection. Notably, vervet Aβ shares 100% sequence identity with human Aβ, and forms aggregates in the brain with age [20]. In captivity, vervets can live into their mid-to-late 20’s [26] and often exhibit age-related declines in cognitive [27–29] and physical function [30, 31] and increases in Aβ deposition [32, 33]. Neuritic plaques are also observed, with immunoreactive phosphorylated tau present in dystrophic neurites [33].

In this study, we measured novel phase-contrast MRI markers of CSF flow dynamics in the central nervous system, and explored relationships of CSF flow with fluid biomarkers associated with AD in 16 female vervet monkeys. Our hypothesis was that diminished CSF flow dynamics in the spinal canal would be associated with fluid biomarkers of AD pathophysiology, reflecting the impairment in waste clearance commonly observed in AD.

## Methods

### Study Subjects

Subjects were members of the Aging Vervet Cohort which is embedded in ten matrilineal social groups in the Vervet Research Colony [29] at Wake Forest Primate Research Center. They included 27 middle-aged to very old females (10 to 27 years of age; median = 22, IQR=[16.5, 23.5]), comparable to approximately 30 to 90 human years of age. [29] This study included 23 vervet monkeys for the phase-contrast MRI (PC-MRI); however, reliable CSF flow data could not be acquired for 7 animals due to unstable cardiac gating. The strong magnetic gradients of the MRI sequence interfered with the ECG signal, resulting in delays in scan time. Thus, the final sample size was n = 16, aged 9 to 29 years,

### MRI Acquisition

**Figure 1:**
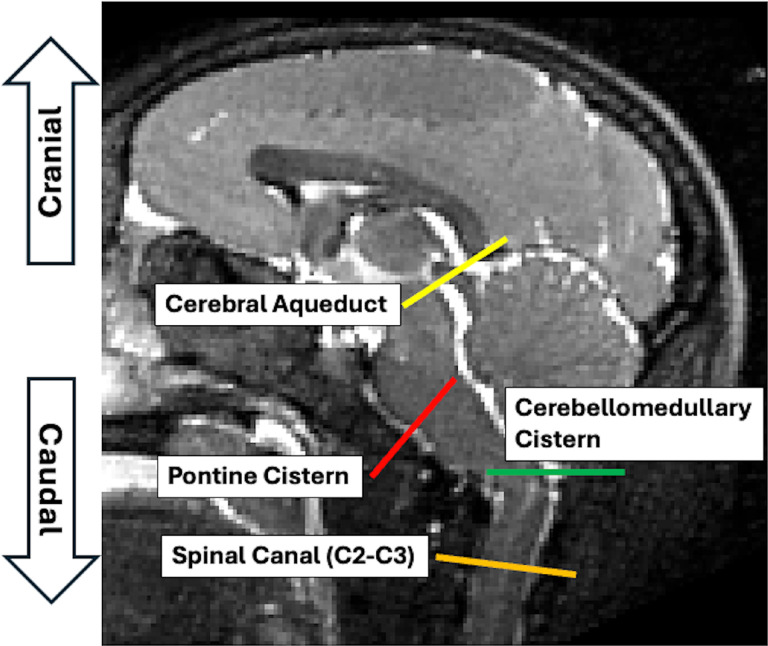
Imaging planes delineated on T2-weighted MRI and the corresponding phase contrast MR images, used for measuring CSF flow at specific regions of interest.

Animals were transferred to the MRI facility, sedated (ketamine HCl, 10–15 mg/kg body weight), and anesthesia was maintained by isoflurane (3% induction, 1.5% maintenance). T1-weighted, T2-weighted and phase-contrast MRIs were acquired using a 3T Siemens Skyra scanner (Siemens, Erlangen, Germany) with a 32-channel pediatric head coil (Litzcage, Doty Scientific, SC) to define the imaging planes for CSF velocity measurements in the subarachnoid space. T1-weighted anatomic images were acquired using a three-dimensional volumetric magnetization-prepared rapid acquisition with gradient echo (MPRAGE) sequence (repetition time [TR] = 2700 ms; echo time [TE] = 3.32 ms; time interval [TI] = 880 ms; flip angle = 8°; 192 slices, voxel dimension = 0.5×0.5×0.5 mm^3^). To enhance the definition of imaging planes for phase-contrast MRI, sagittal T2-weighted MR images were acquired, covering the central regions of the brain (TR = 3200 ms, TE = 251 ms, flip angle = 120°) with 40 slices and a voxel dimension of 0.5 × 0.5 × 1.0 mm^3^ (Fig. 1). CSF velocity measurements were obtained across four transverse planes in the subarachnoid space, targeting CSF flow in the cerebral aqueduct (associated with CSF production), the pontine cistern (anterior CSF perfusion), the cerebellomedullary cistern (posterior CSF perfusion), and the spinal canal.

To measure CSF flow dynamics (i.e. stroke volume, amplitude and peak flow rate), we employed PC-MRI, where cardiac gating is used to ensure that flow measurements are captured at the same relative time points across multiple heartbeats [34, 35].

Phase-contrast velocity imaging used a gradient echo sequence with a TR of 23.72 ms, TE of 7.29 ms, and a flip angle of 10°. The voxel dimension for the single slice phase-contrast imaging was 0.47 × 0.47 × 3.1 mm, with a field of view (FoV) of 180 × 180 mm. Retrospective cardiac gating enabled the capture of 40 distinct cardiac phases per heartbeat, synchronized via electrocardiogram (ECG), with a nominal interval of 453 ms. Pediatric ECG electrodes (Invivo Quadtrode Neonatal ECG Electrodes, Philips Healthcare, Netherlands) were positioned with one electrode on the left upper chest near the left nipple. During CSF flow imaging, reliable flow signals were challenging to acquire at the cerebral aqueduct and pontine cistern, likely due to slow CSF flow and the limited spatial resolution for the small vervet brain. Although CSF flow signals at the cerebellomedullary cisterns were quantifiable, we opted to use the more reliable signal obtained at the spinal canal, specifically at the C2–C3 cervical vertebrae. For CSF flow measurements in the spinal canal, the phase encoding velocity (VENC) was set to 10 cm/s based on our test scans to find the optimal VENC with three animals, with a single average (NEX = 1) to maintain acquisition efficiency.

### MRI Processing

**Figure 2:**
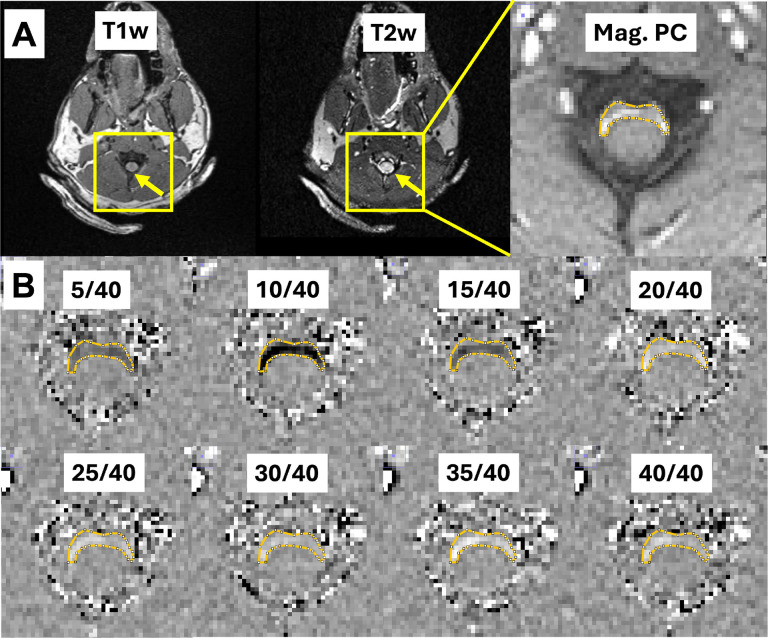
A: T1-weighted, T2-weighted and magnitude image of phase-contrast MRIs. Yellow boxes indicate the field of view for magnitude image of PC-MRI (Mag. PC). Orange line indicates the spinal canal associated with fast CSF flow during the cardiac cycle demonstrating bright signals in magnitude image. B: Corresponding phase images of phase-contrast MRI during the cardiac cycle for a representative animal. Numbers on the top indicate distinct cardiac phases per heartbeat. The whole cardiac cycle consists of 40 phases during the phase-contrast MRI scan.

To estimate the CSF flow velocity profiles at the spinal canal during the cardiac cycle, phase unwrapping [36] and background correction were automatically performed within the scanner’s workflow using the Syngo E11 software before subsequent postprocessing. Voxels of interest were identified from magnitude images of PC-MRI by an experienced biomedical engineer (JK), and these were reviewed by a neuroradiologist (CTW) (Fig. 2).

**Figure 3:**
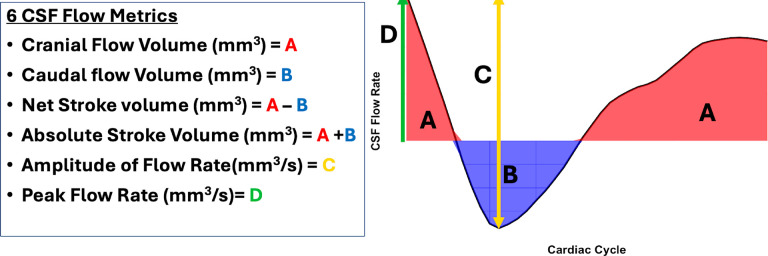
Six metrics to measure CSF flow dynamics in the subarachnoid space during the cardiac cycle.

CSF flow velocity within the spinal canal was estimated by multiplying the phase image by the VENC and dividing by π. The CSF flow rate was then calculated by multiplying the cross-sectional area of the spinal canal by the CSF velocity for each participant. Six dynamic CSF flow metrics were calculated over the cardiac cycle for each participant (as shown in Fig. 3): cranial flow volume, caudal flow volume, net stroke volume, absolute stroke volume, amplitude of flow rate, and peak flow rate.

### Fluid Sample Collections and Biomarker Analyses

We tried to collect CSF and blood samples at the same time ~ 25–30 days prior to MRI studies and processed as previously described [17]. However, due to the delay in biomarker analyses, the time from fluid sample collection to MRI was 101 ± 246 days in the selected dataset. Subjects were sedated with ketamine and maintained in a lateral recumbent position, and CSF samples were collected by inserting a 22-gauge needle percutaneously into the cisternal space Approximately 1–1.5 cm^3^ of spinal fluid was obtained and frozen in 200 ul aliquots at − 70°C until analysis. Aβ_1–42,_ Aβ_1–40,_ and Aβ_1–38_ were measured in first-thawed CSF or plasma using Mesoscale Discovery V-PLEX Plus Aβ Peptide Panel 1 (6E10) (Cat# K15200G) kits, S-PLEX NHP Tau (pT181) Kit (Cat# K156AGMS), as described by the manufacturer. CSF and plasma NfL and GFAP were measured using an in-house duplex assay employing MSD R-PLEX antibody sets for GFAP and NfL paired with U-PLEX Development Pack 2-Assay SECTOR plates and assayed using varying modifications to the MSD standard instructions. Mean coefficient of variation (CV) across all CSF samples was £ 3% for Aβ_1–42_ and Aβ_1–40_, and NfL, 3.5% for GFAP, and 5.5% for pTau181. For plasma, sample CVs were < 3.1% for Aβ_1–42_ and Aβ_1–40_, < 2.4% for NfL, and < 5.1% for pT181. All of the above assays have been validated in vervet and macaque samples internally.

### Statistical Analyses

A combination of correlation, linear regression, and regularization analyses were conducted to test the hypothesis that diminished CSF flow in the spinal canal are associated with biomarkers of impaired clearance in the brain. To examine partial correlations between CSF flow dynamics (including net stroke volume, cranial flow volume, caudal flow volume, absolute stroke volume, flow amplitude, and peak flow), age, and CSF/plasma biomarkers (NfL, GFAP, Aβ42, Aβ40, Aβ42/40 ratio, and p-tau181), the *pcorr* function in the R statistical package (version 4.4.0: http://r-project.org) was used. This approach allowed us assessment of the associations between MRI markers and AD-related fluid biomarkers while adjusting for age.

Given the potential interaction between age-related tissue atrophy and CSF flow dynamics in the subarachnoid space, we conducted linear regression analyses using the *lm* function in R package, including age as a covariate. Statistical significance was determined using a threshold of p < 0.05. To address the potential issue of collinearity between age and CSF flow metrics, we adopted a residualization approach was adopted. To assess the relationship between CSF flow metrics and fluid biomarkers independently of age, we used residuals of each CSF flow metric with respect to age to create age-adjusted components. This allowed us to isolate the unique age-independent contributions of CSF flow dynamics to individual phenotypes.

## Results

### CSF Flow Profiles during the Cardiac Cycle

**Figure 4:**
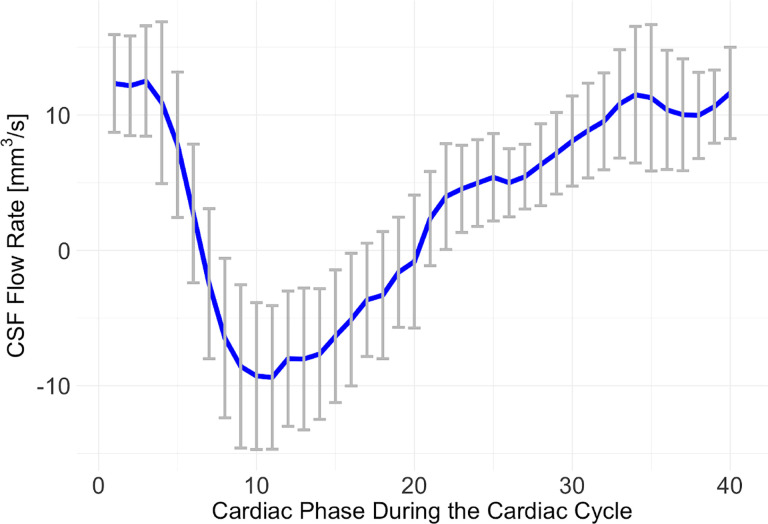
The mean and standard deviations of CSF flow rate at the spinal canal during the cardiac cycle for 16 animals.

Figure 4 demonstrates the averaged CSF flow rate and standard deviations during the cardiac cycle for 16 vervet monkeys at the voxels of interest as shown in Fig. 2. These results demonstrate that CSF flow in the subarachnoid space moves cranially during diastole and caudally during systole, with a delay of approximately 6–7 time points considering zero CSF flow rate between 6th and 7th cardiac phases. Over the cardiac cycle, the cranial CSF flow volume (225 ± 65.7 mm^3^/s × Cardiac Phase) is approximately 2.5 times greater than the caudal flow volume (90 ± 53.1 mm^3^/s × Cardiac Phase), supporting the net movement of CSF into the perivascular space or lymphatic vessels.

### Correlation Between Fluid Biomarkers and PC-MRI Markers

**Figure 5:**
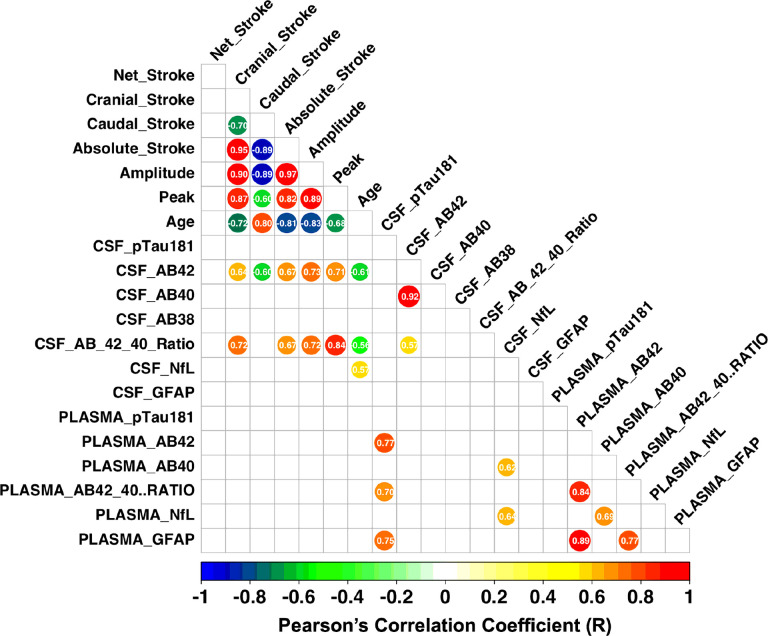
Correlations between CSF flow metrics and fluid biomarkers. Only statistically significant (p <0.05, two-sided tests) correlations were represented.

CSF biomarkers showed significant associations with CSF flow metrics, whereas plasma biomarkers did not. Figure 5 presents the correlation matrix between MRI markers and CSF biomarkers, where PC-MRI markers include six CSF flow metrics. Among the CSF flow metrics, all except the net stroke volume were strongly associated with age (R= −0.68 to −0.83), CSF Aβ42 (R = 0.60 to 0.73), and the Aβ42/40 ratio (R = 0.57 to 0.84), highlighting age-related changes in CSF flow circulation within the central nervous system. Caudal flow volume was inversely correlated with other flow metrics, as expected. Cranial flow volume decreased with age (R= −0.72), while negative stroke volume increased with age (R = 0.80), suggesting a reduction in CSF perfusion into clearance systems with aging. High correlations were observed among CSF flow metrics, as they were derived from the same velocity profiles during the cardiac cycle. Further analysis revealed that CSF Aβ42 and the Aβ42/40 ratio were correlated with CSF flow metrics, with the strongest associations observed for amplitude (R = 0.73) and peak CSF flow (r = 0.84), respectively. Both biomarkers were also associated with age, with amplitude (R = −0.64)) and peak CSF flow (R= −0.56) showing age-related effects. These findings suggest that CSF amyloid pathophysiology is influenced by both CSF flow dynamics and age. In contrast, other biomarkers, including CSF pTau181, Aβ40, Aβ38, NfL, and GFAP, were not associated with CSF flow metrics. Plasma biomarkers were not associated with CSF flow metrics. Full correlation coefficients and p values were reported in the Supplementary Table 1.

### Age-adjusted Effects

Since both age and CSF biomarkers (Aβ42 and Aβ42/40 ratio) were associated with CSF flow metrics in Fig. 5, we conducted partial correlation and residualization analyses to investigate the relationship between CSF flow metrics and CSF biomarkers while controlling for the influence of age (Table 1). The partial correlation analysis revealed a strong association between peak CSF flow in the spinal canal and the Aβ42/40 ratio (R = 0.76, p = 0.005). Additionally, other flow metrics, such as net stroke volume, positive stroke volume, and amplitude, showed moderate associations with CSF biomarkers. These findings suggest that while impaired CSF flow in the spinal canal is influenced by age, it is also independently associated with amyloid pathophysiology. The residualization analysis further confirmed that higher peak CSF flow, independent of age, is positively associated with the CSF Aβ42/40 ratio (p = 0.005), reinforcing the hypothesis that peak CSF flow dynamics may play a significant role in AD pathophysiology. On the other hand, the CSF Aβ42 was not associated with CSF flow metrics after controlling for the age.

**Figure 6:**
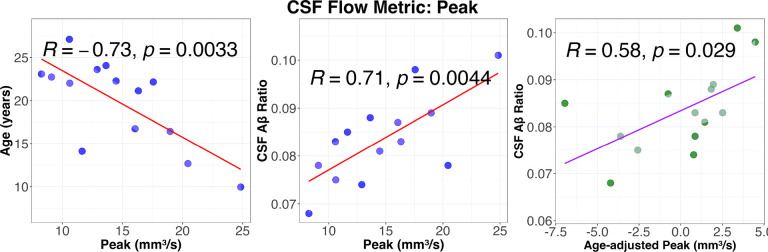
Correlations between CSF flow peak and age and CSF Ab Ratio. Peak flow is negatively correlated with Age (Left,) while positively correlated with CSF Ab Ratio (Middle) and correlation between age-adjusted peak flow rate and CSF Ab Ratio (Right). Correlation coefficients can be different from Figure 5 because they were estimated from residual instead of raw data.

Figure 6 scatter plots between CSF flow metric (Peak), age and CSF Ab ratio. As observed in Fig. 5, age was negatively associated with flow metrics while CSF Ab ratio was positively correlated, indicating that CSF flow perfusion in the spinal canal decreases with age and the decreased CSF flow perfusion is associated with decreased CSF Ab ratio (progression of AD pathophysiology). Plots for other CSF flow metrics were included in Supplementary Fig. 1.

## Discussion

In this study, we utilized the Wake Forest Alzheimer’s Disease Research Center (ADRC) Aging Vervet Cohort to evaluate relationships between CSF flow dynamics in the subarachnoid space and fluid markers of AD pathophysiology. We assessed the feasibility of employing novel MRI markers quantifying CSF flow dynamics in the spinal canal as indicators of amyloid clearance by examining their correlations with AD fluid biomarkers. Our findings revealed that CSF flow metrics were negatively associated with age and positively associated with the CSF AD biomarkers. Age-adjusted analyses further confirmed that CSF flow dynamics are independently associated with the CSF Aβ42/40 ratio, irrespective of age. These results suggest that CSF flow dynamics in the spinal canal may serve as markers for amyloid clearance in the brain. Moreover, this study demonstrated the value of vervet monkeys as a model for AD, offering insights into disease progression related to amyloid clearance from middle to older age.

This study strategically included vervet monkeys spanning a wide age range, from middle to older age, corresponding to approximately 30–90 years in humans. As anticipated, CSF biomarkers in our study showed age-related changes: the CSF Aβ42/40 ratio and Aβ42 levels were negatively associated with age [37, 38], while CSF NfL levels increased with age [39, 40]. Similarly, CSF flow dynamics in the spinal canal exhibited significant age-related changes. Cranial CSF flow and absolute stroke volume decreased with age, while caudal CSF flow decreased, collectively indicating diminished CSF perfusion in the subarachnoid space. This reduction in perfusion suggests a diminished supply of CSF volume into the perivascular space, potentially impairing interstitial clearance by reducing ISF flow [4] and the drainage of ISF and solutes along the basement membranes of capillaries and arteries [41]. Efficient clearance also relies on the transport of waste from the subarachnoid space into meningeal lymphatic vessels [9, 11, 12, 42, 43], underscoring the interconnected roles of CSF circulation and lymphatic drainage. The observed decline in clearance with aging may serve as a key factor facilitating Aβ deposition in the brain [44], further relating impaired CSF dynamics to AD pathophysiology. Interestingly, net stroke volume in the spinal canal was not associated with age in our study. This apparent discrepancy may stem from the low signal-to-noise ratio of PC-MRI, which has limited spatial resolution relative to the sizes of the brain and spinal canal in vervet monkeys. Consequently, individual velocity profiles in the spinal canal showed relatively large standard deviations during the cardiac cycle (as illustrated in Fig. 3), which may have introduced errors in net stroke volume measurements. These findings suggest that caution is warranted when interpreting the relationship between net stroke volume and CSF flow metrics in this study.

While CSF Aβ42/40 ratio significantly correlated with both age and CSF flow metrics, plasma Aβ42/40 ratio and other plasma markers such as p-tau181 showed no such correlation. This is not surprising given the proximity of CSF flow and CSF biomarkers. Plasma biomarkers are cost-effective and minimally invasive to aid in the diagnosis of AD, but to date they do not have the resolution of predictive power of gold standard CSF markers.

Our findings on the relationship between AD fluid biomarkers and CSF flow metrics provide valuable insights into the role of anti-amyloid therapy and early intervention strategies. Enhanced CSF flow in the cranial direction during the cardiac cycle may promote CSF perfusion into perivascular spaces, facilitate ISF bulk flow via the glymphatic system, and support the clearance of CSF into lymphatic vessels at the meninges. As shown in Supplementary Fig. 1, our study highlights that younger animals exhibit superior CSF flow dynamics, suggesting that age-related declines in clearance efficiency may contribute to amyloid accumulation in the brain parenchyma. Maintaining healthy CSF flow dynamics in the central nervous system is critical as an early intervention approach. Simple lifestyle modifications, such as changes in breathing patterns or regular exercise, have shown potential to improve CSF flow. For instance, forced breathing alters the dominant frequency of CSF and venous flow spectra, shifting it toward the respiratory component. It also increases average CSF flow magnitude across spinal and intracranial regions [45]. Yogic breathing practices increased 16–28% in the power and velocity of CSF flow compared to spontaneous breathing. Deep abdominal breathing elicited the most significant increases in CSF oscillation [46].

Therapies such as Aducanumab [47] and Lecanemab [48] target Aβ aggregates by binding to soluble, oligomeric, or fibrillar forms of Aβ [49]. After these aggregates are broken down, their effective clearance relies on CSF flow dynamics to transport soluble Aβ from the brain’s ISF to lymphatic drainage pathways or to sites where immune cells, such as meningeal macrophages, actively degrade these fragments [50, 51]. Efficient CSF circulation ensures that Aβ aggregates reach lymphatic drainage pathways, such as the nasal lymphatics or dural lymphatics and are exposed to immune clearance mechanisms in meninges or perivascular regions. CSF flow dynamics could serve as a biomarker to identify patients who might benefit most from anti-amyloid therapies, especially when paired with interventions designed to enhance clearance. Regular monitoring of CSF flow metrics alongside PET and plasma biomarkers can also help evaluate the efficacy of these therapies. For instance, a reduction in amyloid burden, as observed through PET or fluid biomarkers, combined with stable or improved CSF flow dynamics, may indicate a successful therapeutic response.

This study focused exclusively on female subjects, motivated by the higher prevalence of AD in women compared to men [52, 53]. We did not investigate apolipoprotein E-e4 allele (APOE4) effects, a strong genetic risk factor for AD, as all NHPs studied to date carry only variants of APOE4 [54]. Consequently, the clearance behaviors observed in these NHP models are more likely similar to APOE4 carriers than APOE4 non-carriers in humans. With aging, vervets develop early AD-like neuropathology that includes increasing amyloid plaques, decreasing AB42 in CSF, decreases in brain volume, and declines in cognitive function. These, naturally occurring AD pathologies are like those observed in the early stages of AD neuropathogenesis, a time in which potential therapies may be most efficacious.

Another limitation of this study was sample size. This study included 23 vervet monkeys for PC-MRI imaging; however, reliable CSF flow data could not be acquired for 7 animals due to unstable cardiac gating. The strong magnetic gradients of the MRI sequence interfered with the ECG signal, resulting in delays in scan time. However, despite the small cohort, we observed a strong linear relationship between CSF flow metrics and AD biomarkers, supporting our hypothesis that CSF flow dynamics are linked to amyloid clearance at earlier stages of disease progression. [52–56]

## Conclusion

Diminished CSF perfusion in the central nervous system, as quantified by phase-contrast MRI, was found to be negatively correlated with age and positively correlated with the CSF Aβ42/40 ratio in the aging vervet cohort at Wake Forest. Age-adjusted analyses revealed moderate correlations between CSF flow dynamics and the Aβ42/40 ratio, supporting the hypothesis that impaired CSF flow may serve as a valuable clearance biomarker indicative of AD-like pathology. These findings highlight the potential role of CSF flow dynamics as an early marker for AD risk and progression, reflecting age-related declines in clearance efficiency. Future clinical studies should expand on these results by including participants with different APOE genotypes (both e4-carriers and non-carriers). Additionally, longitudinal investigations could evaluate whether interventions targeting CSF flow dynamics improve clearance efficiency and delay the onset of AD-related pathology.

## Supplementary Files

This is a list of supplementary files associated with this preprint. Click to download.

- SupplementaryDocs.docx

## Figures and Tables

**Table 1:** Summary of statistics for partial correlation and residualization analyses between CSF flow metrics and CSF Ab42/40 Ratio.

CSF Flow Metrics		Net Stroke	Cranial Stroke	Caudal Stroke	Absolute Stroke	Amplitude	Peak
Partial Correlation	R	0.556	0.556	−0.048	0.450	0.560	0.755
	p	0.061	0.060	0.883	0.142	0.059	**0.005** **
Residualization	Estimate	0.0028	0.0031	−0.0005	0.0019	0.0009	0.0017
	Std. Error	0.0013	0.0015	0.0030	0.0012	0.0004	0.0005
	t value	2.114	2.117	−0.151	1.592	2.135	3.639
	Pr(>|t|)	0.061	0.060	0.883	0.143	0.059	**0.005** **

## Data Availability

MRI and fluid biomarker data can be provided upon reasonable request to jeokim@wakehealth.edu.

## Abbreviations

Aβ: amyloid-beta
AD: Alzheimer’s disease
ADRC: Alzheimer’s Disease Research Center
APOE4: apolipoprotein E-e4 allele
BBB: blood-brain barrier
CSF: cerebrospinal fluid
CV: coefficient of variation
ECG: electrocardiogram
FoV: field of view
IPAD: intramural peri-arterial drainage
ISF: interstitial fluid
MPRAGE: magnetization-prepared rapid acquisition with gradient echo
NHPs: nonhuman primates
PC-MRI: phase-contrast MRI
TE: echo time
TI: time interval
TR: repetition time
VENC: encoding velocity
